# Towards Robust Supervised Pectoral Muscle Segmentation in Mammography Images

**DOI:** 10.3390/jimaging10120331

**Published:** 2024-12-22

**Authors:** Parvaneh Aliniya, Mircea Nicolescu, Monica Nicolescu, George Bebis

**Affiliations:** Computer Science and Engineering Department, College of Engineering, University of Nevada, Reno, Main Campus, Reno, NV 89557, USA; mircea@unr.edu (M.N.); monica@unr.edu (M.N.); bebis@unr.edu (G.B.)

**Keywords:** breast cancer mammography, pectoral muscle, INbreast, CBIS-DDSM, MIAS, deep learning, supervised training

## Abstract

Mammography images are the most commonly used tool for breast cancer screening. The presence of pectoral muscle in images for the mediolateral oblique view makes designing a robust automated breast cancer detection system more challenging. Most of the current methods for removing the pectoral muscle are based on traditional machine learning approaches. This is partly due to the lack of segmentation masks of pectoral muscle in available datasets. In this paper, we provide the segmentation masks of the pectoral muscle for the INbreast, MIAS, and CBIS-DDSM datasets, which will enable the development of supervised methods and the utilization of deep learning. Training deep learning-based models using segmentation masks will also be a powerful tool for removing pectoral muscle for unseen data. To test the validity of this idea, we trained AU-Net separately on the INbreast and CBIS-DDSM for the segmentation of the pectoral muscle. We used cross-dataset testing to evaluate the performance of the models on an unseen dataset. In addition, the models were tested on all of the images in the MIAS dataset. The experimental results show that cross-dataset testing achieves a comparable performance to the same-dataset experiments.

## 1. Introduction

Breast cancer is one of the main cancer types in the female population, with a high mortality rate. Mammograms are images taken from two views of a compressed breast region. These views are craniocaudal (CC) and mediolateral oblique (MLO) views. CC is the view in which the breast is compressed horizontally, and in the MLO view, the compression is diagonal. Mammography images are the most commonly used tool for breast cancer screening due to their availability and lower cost. Therefore, the development of automated cancer detection methods for these images is of high importance due to the benefits they bring to patients by increasing survival chances by detecting the abnormalities accurately in the early stages [1].

While current methods have improved the performance considerably, several challenges hinder the performance of these methods for mammography images. For instance, in the MLO view, a portion of the pectoral muscle is usually visible in the final image. Pectoral muscles, fibroglandular tissue, and abnormalities all appear as brighter regions compared to the fatty tissues in the images. Therefore, detecting abnormalities becomes more challenging in cases with high breast tissue density or in the presence of the pectoral muscle. This emphasizes the need for approaches to address the challenges of abnormality detection in high-density cases and removal of the pectoral muscle in the images.

This paper targets the latter problem, which is also highly important for automated density estimation. Due to the fact that the segmentation mask of the pectoral muscle is not normally available in the publicly available datasets or in the examinations for breast cancer screening in current practice in clinical settings, most of the current pectoral muscle removal approaches are based on traditional machine learning approaches. As the muscle’s location, shape, density, and position vary between the images, the performance of these methods is limited. Ideally, utilizing deep learning-based approaches would help mitigate these hurdles to a great extent if annotations for the pectoral muscle were available.

We argue that providing annotations even for several datasets will enable researchers to train supervised pectoral muscle removal and utilize them for new unseen datasets. Hence, following the work of Aliniya et al. [2], we provide pectoral muscle segmentation for three benchmark datasets, INbreast [3], MIAS [4] and CBIS-DDSM [5]. The segmentation masks are available at https://github.com/Parvaneh-Aliniya/pectoral_muscle_groundtruth_segmentation, accessed on 1 December 2024. We separately trained AU-Net [6], a widely used segmentation method for mammography images, on the datasets. Same-dataset and cross-dataset tests were used to validate the proposed method. In same-dataset tests, train and test sets belong to one of the datasets; in the cross-dataset experiments, the train and test sets are from two different datasets. The models achieve high accuracy for both same-dataset and cross-dataset experiments.

Pectoral muscle removal methods are beneficial for tasks such as density estimation in which the presence of pectoral muscle decreases the accuracy of the estimation when the muscle is wrongly detected as dense tissue. Moreover, they could be used as a preprocessing step in segmentation and classification tasks. In addition, the pectoral muscle removal methods could also improve the performance of multi-view approaches that use both MLO- and CC-view images as input.

The contributions of this paper are three-fold:

- Generating the segmentation masks of pectoral muscle for INbreast, MIAS, and CBIS-DDSM datasets.
- Training pectoral muscle removal models using the AU-Net architecture separately for INbreast and CBIS-DDSM datasets.
- Evaluating the models by same-dataset and cross-dataset testing to measure the generalizability of the supervised trained models on the same and new datasets.

In the following, we first review the literature on pectoral muscle removal and segmentation methods for mammography images and then present the proposed method. Finally, the experimental results section provides the results of the experiments and comparisons with state-of-the-art methods.

## 2. Related Work

In this section, we provide a review of the previous methods proposed to tackle the pectoral muscle removal task [7,8,9,10,11] and a brief review of the segmentation method with a focus on those proposed for mammography images. Some methods consider the task as segmentation, and others use the phrase “pectoral muscle removal”. In the paper, we use both phrases according to the context. As it is a trivial task for radiologists to exclude the pectoral muscle visually while reading the mammograms, the segmentation of the pectoral muscle is not available in images in most datasets; hence, to the best of our knowledge, all of the proposed methods are traditional machine learning-based methods. These methods, in general, aim to use the appearance of the muscle and its location (after prepossessing to unify the alignment of the breast to be all left or right) to detect and eliminate it.

### 2.1. Pectoral Muscle Removal Methods

According to a recent study [1], thresholding [12,13,14,15] and region growing [16,17,18] are widely used approaches.

The general idea for thresholding is to use the observation that the brightness of the pectoral muscle is generally higher than the neighboring regions; therefore, by eliminating pixels lower than a certain threshold, the region for the pectoral muscle will be extracted. This idea, coupled with the utilization of the orientation of the breast (whether the breast is on the left or right side of the image) and the generic shape of the muscle, has been used in the literature. In this category, Subashini et al. [13] used a thresholding-based approach for pectoral muscle removal, in which they first extracted the rectangle in the image where the pectoral muscle was assumed to be located and then used thresholding within the rectangle to detect the muscle. The height of the rectangle was fixed relative to the image’s height, and its width was selected according to the width of the breast area on top of the image. Tayel et al. [14] proposed an approach that eliminates the need for a predefined region. To this end, they employed the idea of retaining only a region corresponding to the location of the muscle after thresholding. In the same category, Czaplicka et al. [15] proposed using multi-level thresholding, and Shrivastava et al. [19] developed a method using a sliding window for thresholding.

There are several drawbacks to these approaches. First, in many cases, the difference between the brightness of the pectoral muscle and the surrounding pixels is not high enough to lead to an accurate boundary for the pectoral muscle using thresholding. In addition, there are certain artifacts that are overlaid on the pectoral muscle region (such as tape or notes) in some images that introduce errors to the thresholding method. Moreover, the region for the pectoral muscle is not always consistent, so thresholding may lead to sub-optimal results in such cases. Finally, predefined regions for the selection are not generalizable to all the cases, and the pectoral muscle may exceed the region.

The second category of methods for pectoral muscle removal consists of region-growing-based methods. These methods generally start with initial seeds; then, according to certain similarity metrics, they continue adding a new neighboring pixel to a region until a termination criterion is met [16]. Chen et al. [20] proposed using a pixel near the border between the pectoral muscle and the breast tissue (which was approximated) as a starting seed. For the ending threshold value, they used growing thresholding, which stops near the edges of the image. This approach relies on the border of the pectoral muscle being well-defined, which does not apply to many samples, specifically for samples with higher breast tissue density. Instead of approximating the location for the border, Nagi et al. [21] used the approximate location for the pectoral muscle after determining the orientation for the breast to place the initial seed. Then, the region-growing algorithm was applied to the starting point. Maitra et al. [22] introduced several improvements to the previous method by using a triangle that encapsulated the pectoral muscle after flipping the images (to achieve left orientation for all images). For seed selection, they proposed to use the diagonal of a defined rectangle encapsulating the pectoral muscle from top-left to bottom-right. The points in the line that were located inside the triangle were selected as the seeds. In addition, they used new selection criteria based on the minimum, maximum, and average values for the pixels. Priyanka et al. [23] proposed a region-growing method by optimizing the initial seed selection stage. The flooding algorithm was used to grow the region, and the process of adding a new point was conditioned on a predetermined intensity standard.

Aside from the previous methods that aim to use region growing and thresholding, graph-cut [24], Hough Transform [25], line estimation, polynomial fitting, curve estimation [26], k-means [27], active contours [28], and contour growing [29] are also used in several methods. For instance, Dhimann et al. [30] proposed to first blur the image and then apply the Canny edge detector on the image. Finally, the Hough lines were detected from the detected edges, which were further processed to select the best line. This method has a few limitations. First, selecting the degree of blurring as a hyperparameter is challenging, specifically in mammograms where the grayscale range may change from one image to another. In addition, selecting a line as the border of the muscle decreases the detection accuracy. Mahaveera et al. [31] proposed a cluster-based method that used intensity as the value for clustering. Then, the connected-components-labeling algorithm was utilized to differentiate between the muscle and breast regions (the assumption about the location of the masses was used in this stage). Finally, they refined the region to improve the results. The main drawback of this method is that in more challenging cases in which the boundary between the muscle and the breast region is unclear (or the diversity of the pixel values is high in the breast region), a considerable number of pixels might be misplaced. Chen et al. [32] developed a method that relies on image binarization to help distinguish between bright and darker regions (as the pectoral muscle is usually brighter than the surrounding regions). A Canny edge detector was applied to the resulting image, and the edges were improved with interpolation. This method is sensitive to noise in the images and unclear borders between the muscle and the breast tissue.

### 2.2. Segmentation Methods

In recent years, many methods have been proposed for segmentation in mammography images and other domains. In this section, we focus on reviewing the segmentation methods, specifically for the segmentation of masses in mammography images. As masses are one of the dominant abnormalities in the breast, most of the segmentation methods for mammography images focus on this group.

The authors of [33,34] were among the first to design a segmentation method using deep learning. U-Net [35] is a pioneering work in deep learning-based segmentation methods for medical applications. The fully convolutional network (FCN) [33] introduced a network with skip connections and end-to-end training for segmentation tasks. U-Net extended the core idea of FCN by proposing a symmetric encoder–decoder network that differed from FCN in that the skip connections were more present throughout a network with a symmetric structure in the encoder and decoder parts. In addition, instead of summation, U-Net utilized concatenation for the feature maps and further processed the output of the concatenation.

The methods up to this point were generic segmentation methods, focusing on improvement of the performance. However, to achieve the best performance for specific tasks, such as applications in mammography images, it is vital to take the specific characteristics of the input and the desired output into consideration. Hence, following the success of U-Net and FCN, in recent years, studies [36,37,38,39,40,41,42,43,44,45] in the medical imaging domain have exceeded the performance limits of segmentation through the adaptation and advancement of these approaches. These approaches have been proposed for a variety of medical images, such as images for pelvic organs [36] and gland segmentation [44]. For instance, Drozdzal et al. [46] explored the idea of creating a deeper FCN by adding a short skip connection to the decoder and encoder paths in order to improve the performance of segmentation for biomedical images. Zhou et al. [45] developed more sophisticated skip connections to create more semantically compatible features before merging the feature maps from the contracting and expanding paths. This method was tested on images of liver, colon polyp, and cell nuclei.

Hai et al. [47] improved the design on UNet while considering the challenging features of the mammography data, such as the diversity of shapes and sizes. To this end, they utilized an Atrous Spatial Pyramid Pooling (ASPP) module in the transition between the encoder and decoder paths. The ASPP block consisted of 1×1 conv plus three atrous convolutions [48] with sample rates of 6, 12, and 18; the outputs for these layers were concatenated and fed into a 1×1 conv. FC-DenseNet [49] was selected as the backbone of the method. Shuyi et al. [50] is another U-Net-based approach based on the idea of utilizing densely connected blocks for mass segmentation in mammography images. In the encoder, the path is constructed from densely connected CNNs [51]. In the decoder, gated attention [39] modules are used when combining high- and low-level features, allowing the model to focus more on the target. Another line of research within the scope of multi-scale studies is [52], in which the generator is an improved version of U-Net for mass segmentation. Multi-scale segmentation results were created for three critics with identical structures and different scales in the discriminator. Ravitha et al. [53] developed an approach to use the error of the outputs of intermediate layers (in both encoder and decoder paths) relative to the ground truth labels as a supervision signal to boost the model’s performance. In every stage of the encoder and decoder, attention blocks with upsampling were applied to the outputs of the block. The resulting features were linearly combined with the output of the decoder and incorporated into the objective criterion of the network to enhance the robustness of the method.

Sun et al. [6] introduced an asymmetric encoder–decoder network (AU-Net). In the encoder path, ResBlocks (three conv layers with a residual connection) were used; in the decode path, basic blocks (including two conv layers) were utilized. The main contribution of the paper was a new upsampling method. In the new Attention-guided Upsampling (AU) Block, high-level features were upsampled through dense and bilinear upsampling. Then, the low-level features were combined with the output of dense upsampling through element-wise summation. The resulting feature maps from the previous step were concatenated with the output of bilinear upsampling and were fed into a channel-wise attention module. Finally, the input of the channel-wise attention module was combined with its output by channel-wise multiplication. AU-Net [6] was the baseline method in this study.

## 3. Materials and Methods

This section presents the process for segmenting pectoral muscles, followed by the proposed training scheme for pectoral muscle removal in mammography images.

### 3.1. Ground Truth Generation for Pectoral Muscle

LabelMe [54] was used to segment the pectoral muscle, in which polygons were fitted to the pectoral muscle for the MLO images in INbreast, CBIS-DDSM, and MIAS datasets. For images with higher density or lower visibility of the boundaries of the pectoral muscle, the portion of the muscle that was clearly distinguishable from the breast tissues was selected. The segmentation masks were generated in JSON and image formats with two classes, the pectoral muscle and background (the remaining breast tissues and image background).

### 3.2. Datasets and Preprocessing

INbreast contains a group of 150 cases with 410 high-resolution CC- and MLO-view images. The pectoral muscle masks for all of the MLO-view images in the INbreast dataset (except for several images in which the pectoral muscle was not presented or distinguishable) were provided in this study and used for the experiments. For the validation, due to limited samples, 5-fold cross-validation was used with the random division of 80%, 10%, and 10% for train, validation, and test sets, respectively. It should be noted that while the pectoral muscle masks were presented in the original INbreast dataset for consistency with two other datasets in the labeling process, we provided the labeling for INbreast as well.

CBIS-DDSM is an enhanced subset of the DDSM dataset. It consists of 1231 training images and 360 test images. CBIS-DDSM is commonly used for the segmentation task in the literature [6], and we provided the pectoral muscle segmentation masks for all of the MLO-view images in the CBIS-DDSM dataset. The standard split for the train and test sets was used in this study. For the validation set, 10% of the training set was randomly sampled.

The MIAS dataset contains only MLO-view images; therefore, it is widely used in proposed methods for the pectoral muscle removal task. MIAS has a total of 322 images. Providing the pectoral muscle segmentation masks is important for research in this domain, so we also included the segmentation masks for all the images in the MIAS dataset (unless the muscle was not visible in the images). We used MIAS for cross-dataset testing using models trained on INbreast and CBIS-DDSM datasets.

For all of the datasets, cropping, padding, resizing, and artefact removal were performed as needed.

### 3.3. Pectoral Muscle Segmentation

The main motivation for this study is to provide the segmentation of the pectoral muscle for several datasets, which enables the training of pectoral muscle removal methods that could also be applied to new unseen datasets. The main use-case of these segmentation masks will be in the removal of pectoral muscle in the preprocessing step for tasks such as the classification of images (for instance, benign/malignant), segmentation of the masses and other abnormalities, and density estimation. To use the segmentation masks for a new dataset, first, a segmentation model should be trained using the provided muscle segmentation masks, and then, the model can be used for the segmentation of the pectoral muscles in a new dataset.

Given the segmentation masks of the pectoral muscles, we proposed to use deep learning-based methods for pectoral muscle segmentation. To this end, we selected AU-Net, a method for segmenting mammography images. AU-Net [6] is an improved version of the U-Net [35] in which the encoding and decoding paths are not symmetrical. ResUnit and the basic decoder proposed in the AU-Net were used for the encoder and the decoder. The details of the novel idea of AU-Net, the AU Block, were presented in the AU-Net approach [6]. A binary cross-entropy loss function was used in the proposed method.

For the training stage, we used early stopping, with a learning rate of 0.00001, and Adam optimizer. The models were trained on an RTX 4090 GPU. Cross-validation was utilized for the INbreast dataset. We implemented the method using TensorFlow. After training on the INbreast, CBIS-DDSM, and a combination of both datasets, the models were used for the same- and cross-dataset tests to evaluate their performance.

### 3.4. Evaluation Metrics

Dice Similarity Coefficient, (DSC, Equation (1)), sensitivity (Equation (2)), and accuracy (Equation (3)) were selected as the evaluation metrics in all of the experiments due to the complementary information they provided. As pectoral muscles occupy a small portion of the images, using accuracy alone would not have been an informative means of evaluating the method. Hence, using an additional metric, such as sensitivity, allowed us to measure the false negative rate. DSC measures the ratio of the correctly predicted positive pixels over the number of positive areas in both the ground truth and the prediction mask, considering the false positive rate in the calculations alongside false negatives. Therefore, we also included DSC in the evaluation metrics.

(1) $$DSC=\frac{2TP}{2TP+FP+FN}$$

(2) $$Sensetivity=\frac{TP}{TP+FN}$$

(3) $$Accuracy=\frac{TP+TN}{TP+TN+FN+FP}$$

Here, TP, TN, FP, and FN represent true positive, true negative, false positive, and false negative rates, respectively.

## 4. Experimental Results

In this section, an evaluation of the results of the same- and cross-dataset experiments, as well as a comparison with previous methods, is presented.

### 4.1. Results for INbreast and CBIS-DDSM

**Figure 1:**
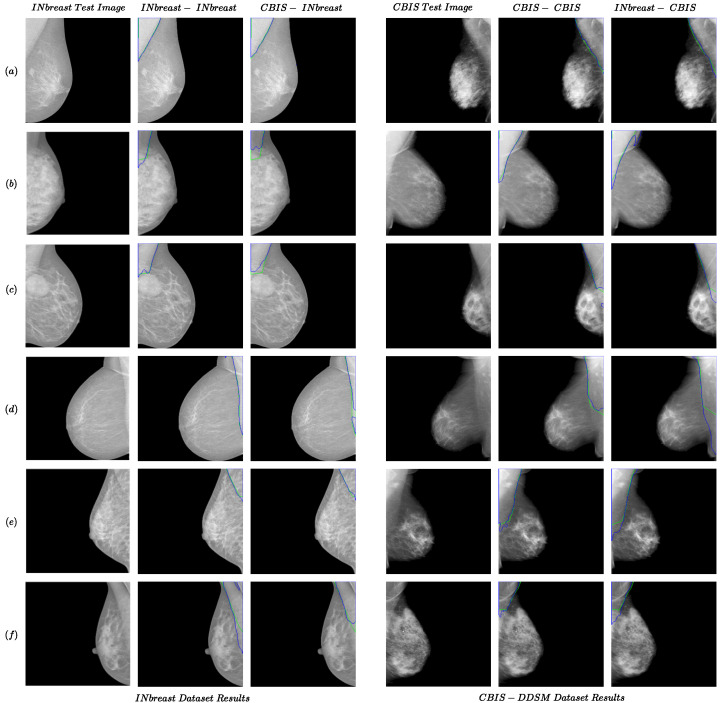
Examples of the performance of the proposed method for the same- and cross-dataset tests for INbreast and CBIS-DDSM datasets. Each row from (**a**–**f**) presents two examples from INbreast and CBIS-DDSM datasets. The green and blue colors present boundaries for the ground truth and predicted segmentation.

The results for the INbreast and CBIS-DDSM datasets are presented in Table 1. The following format was used for the names of the experiments: “train dataset name—test dataset name”. As shown in the first two rows of the table, the models trained with CBIS-DDSM generally outperformed those trained with INbreast in same-dataset experiments. The pectoral muscles generally have higher pixel intensities in the CBIS-DDSM (compared to the rest of the breast regions) compared to the INbreast. In addition, more training images are available in the CBIS-DDSM. These could be the reasons for the better performance of the model on the CBIS-DDSM dataset. Some of the results for same-dataset experiments are shown in Figure 1. For convenience, we used ‘CBIS’ instead of ‘CBIS-DDSM’ in the figures and tables. As shown in the INbreast-INbreast and CBIS-CBIS columns from Figure 1, the power of extracting the features from data automatically in a deep learning-based model enabled our methods to overcome challenges that would impact traditional methods. For instance, the extra line in the pectoral muscle area (Figure 1d (INbreast) and Figure 1b (CBIS)), the ambiguity of the boundary (Figure 1b,e (INbreast) and Figure 1f (CBIS)), presence of a mass in the muscle area (Figure 1c (INbreast)) would have posed challenges for the traditional method; however, the proposed method was able to perform well in these cases.

### 4.2. Results for Cross-Dataset Tests

**Figure 2:**
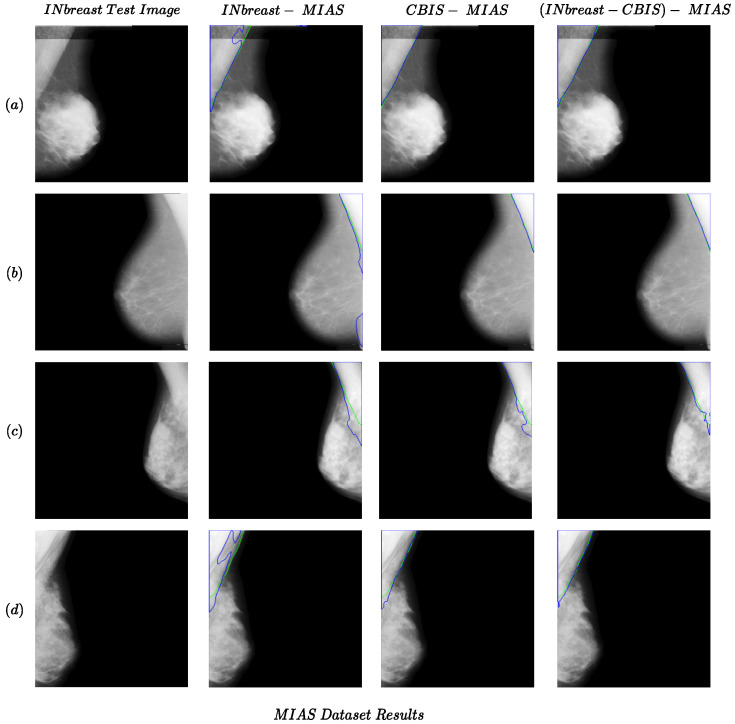
Examples of the performance of the proposed method for cross-dataset tests for the MIAS dataset as the test set. Each row (**a**–**d**) presents results for one sample in the MIAS dataset. The green and blue colors present boundaries for the ground truth and predicted segmentation.

In order to measure the generalizability of the models, we conducted five cross-dataset tests (presented in the last five rows in Table 1). One important aspect of the results for cross-dataset experiments is that when trained or tested on INbreast, while being highly accurate, the performance was generally lower than other cross-dataset experiments. One reason could be the fact that images in these datasets were acquired separately in different conditions; therefore, they may also visually differ. This could be observed in the differences between samples from the INbreast dataset compared to the MIAS and CBIS-DDSM datasets (which seem to be more similar, specifically the brightness of pectoral muscle) in Figure 1 and Figure 2. Therefore, the similarities in the setting in which images were recorded also affect the cross-dataset experiments. With this observation, to make the cross-dataset model more robust, we also trained the model using the combination of the CBIS-DDSM and INbreast for the cross-dataset tests on the MIAS dataset (combined-MIAS row in Table 1). As shown in the last row, the combined-MIAS experiment achieved the best results in the cross-dataset setting. It should be noted that for experiments with the MIAS dataset as the test set, the whole dataset was used.

Some examples of the cross-dataset experiments are shown in Figure 1 (CBIS-INbreast and INbreast-CBIS columns) and Figure 2. The examples of cross-dataset models show comparable performance to the same-dataset models. This confirms the validity of our idea regarding the generalizability of the pectoral muscle removal method proposed in the study. Regarding the MIAS dataset results in Figure 2 and Table 1, the performance of the Combined-MIAS model is better than that of training on each of the INbreast and CBIS-DDSM dataset separately. In Figure 2, all the CBIS-MIAS samples have better results than the INbreast-MIAS.

### 4.3. Comparison with State-of-the-Art Methods

Table 2 compares the proposed method with previous approaches for pectoral muscle segmentation. The proposed method had a superior performance for both same- and cross-dataset experiments compared to the previous methods in terms of accuracy. The values in the second row in the table indicate the number of images used for testing each method. Compared to the best-performing approach among the methods that used all 322 images for testing, the proposed method improved the accuracy by 1.27% on average between three variations. Training a deep learning model enables the method to be more robust for samples that might not align well with the assumptions of traditional methods. It should be noted that accuracy was the shared metric used in the previous methods; hence, we used it as the comparison metric and were not able to include DSC and sensitivity, as they were not available in most of the previous works.

**Figure 3:**
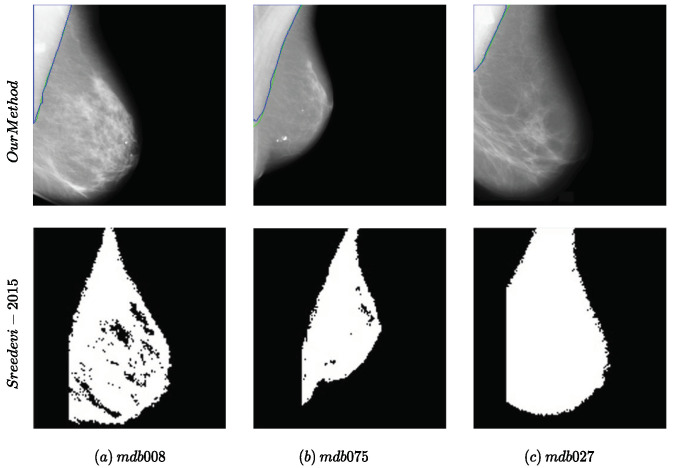
Examples of the performance of the proposed method compared to the method proposed in [7] for the MIAS dataset. The names of the samples in the dataset are mentioned in (**a**–**c**).

Regarding the visual comparison, as neither the implementation nor the full results on the datasets were available, we compared our method with one of the previous works that included visual results with the image ID [7] for the MIAS dataset. The results are presented in Figure 3. Our method achieved better results in comparison with [7] with smoother edges without any further postprocessing.

## 5. Discussion

This study proposed a supervised segmentation method for the pectoral muscle in mammography images. The goal is to use this segmentation in the preprocessing steps for other tasks such as classification, segmentation of the abnormalities, and image registration for multi-view approaches. In mammography images, in some cases, the tissue and pectoral muscle are both visible as they are layered on top of each other. Therefore, in some cases, removing the pectoral muscle might remove the portion of the tissue in the images, which could be harmful for tasks such as density estimation. With this observation in mind, the following approach was used instead of solely focusing on the segmentation of the pectoral muscle in the annotation process. For cases with ambiguous boundaries for the muscle or very low visibility of the muscle, the annotation covered the portion that was mainly and more obviously part of the pectoral muscle to preserve more of the breast tissue in the images. One additional challenge is the diversity of the appearance of the pectoral muscle in the images. When there are limited data for complex appearances, the model’s performances suffer, which could be addressed by utilizing more training data or postprocessing. Moreover, the diversity in the images from the datasets affects the generalizability of the models for new unseen data. The results of cross-dataset tests in this study confirm the need for more diverse datasets in the training process.

**Table 2:** Comparison between the proposed methods for pectoral muscle removal.

Method	[29]	[55]	[56]	[26]	[15]	[20]	[18]	(CBIS)	(INbreast)	(Combined)
Number of Images	322	322	161	322	300	321	322	322	322	322
Accuracy	98.1	92.2	93	96.81	98	97.8	95	99.45	99.03	99.56

## 6. Conclusions

In this study, we provided segmentation masks for INbreast, MIAS, and CBIS-DDSM subset datasets. The segmentation masks provided in this paper will open the door to the use of supervised deep learning-based methods in research on pectoral muscle removal in mammography images. In addition, we trained AU-Net on the datasets separately and achieved an accuracy of 99.55% and 99.61% for the pectoral removal task on INbreast and CBIS-DDSM datasets, respectively. In order to examine the generalizability of these models for new datasets, we also performed cross-dataset tests, which achieved high performance as well. We also tested both models on the entire MIAS dataset, resulting in accuracies of 99.03% and 99.45% for models trained on the INbreast and CBIS-DDSM, respectively. To improve the diversity of the appearance of the samples, a third model was trained on the combination of the INbreast and CBID-DDSM, resulting in an accuracy of 99.56% for the MIAS dataset. The masks provided in this study could be employed for pectoral muscle removal as a preprocessing step in a variety of tasks for mammography images, including classification, segmentation, and density estimation. In addition, the models trained using this information could be utilized for pectoral muscle removal in new unseen data.

## Figures and Tables

**Table 1:** Results for pectoral muscle segmentation for same- and cross-dataset experiments. In the name of the experiments, the first term is the training dataset, and the second is the test dataset.

Train–Test Pair	DSC	Sensitivity	Accuracy
CBIS-CBIS	96.59	96.89	99.61
INbreast-INbreast	95.09	95.54	99.55
INbreast-CBIS	91.77	97.68	99.05
CBIS-INbreast	89.52	82.55	99.13
CBIS-MIAS	94.20	92.26	99.45
INbreast-MIAS	90.64	95.44	99.03
Combined-MIAS	95.39	93.73	99.56

## Data Availability

INbreat, CBIS-DDSM and MIAS datasets are publicly available at https://www.kaggle.com/datasets/tommyngx/inbreast2012 (accessed on 1 January 2023), https://wiki.cancerimagingarchive.net/pages/viewpage.action?pageId=22516629 (accessed on 1 January 2023) and and https://www.repository.cam.ac.uk/items/b6a97f0c-3b9b-40ad-8f18-3d121eef1459 (accessed on 1 January 2023), respectively.
